# Annotations

**Published:** 1891-01-17

**Authors:** 


### Annotations.

**Philip Henry Gosse.**

Mr. Philip Henry Gosse was everybody's "Man of Science." He had nothing of the scentific prig about him. To him the "common objects of the sea-shore," and all the other forms of life which he so ardently studied were matters to be talked of as freely and as naturally as the farmer talks of his ploughing and sowing, or the carpenter of the windows and doors he makes. Prigs are always and everywhere detestable; and none more so than the prigs of science. In the biography which has just been published, Mr. Edmund Gosse says of his father: "He had no notion of striking a happy mean between his impressions of nature and his convictions of religion. If the former offered any opposition to the latter they were swept away." In other words, Mr. P. H. Gosse was the kind of man who felt that though he might misunderstand the nature and causes and sequences of the facts which were outside him he could not misunderstand the authoritative utterances of the moral law within. In its essence that was a profoundly scientific attitude of mind; although we cannot but think that Mr. Gosse erred seriously in confounding the local and temporary religious system which he himself had accepted with the universal religion which begins and ends with "Our Father which art in heaven." Religion existed before there were any objective facts to which it could attach itself; before there was an altar, or an ark, or a law, or a priesthood; and it would continue to exist if all those were swept away, if man remained the same in essential characteristics as he is now. It is as much a part of the essence of man as thought or hearing, or the sense of smell. It can never be banished from the earth until man's nature is changed, or he becomes extinct. Revelations, as they are called, simply make clearer to the human mind both its own nature and the objects not itself which it desires to know, and with which it feels impelled to ally itself. All science is a revelation, in that it makes clear to the mind what the senses previously apprehended with dim uncertainty; and the successive revelations which have been given to man of spiritual and moral things have merely made clearer and more definite what from the first he felt to be right and true. The sum of the revealings which have been given, not only to Jews and Christians, but to Buddhists and Confucians, to Greeks, Romans, and Mohammedans, is this—that Reason was first in the order of things—Reason, including justice and parenthood—and that it will be last. "In the beginning was the Logos . . . . and the Logos was God." With such a religion, which is at once individual and social, Christian and universal, science can never come into conflict. It is when men fail to perceive that God is the parent and the friend of all human beings equally; and that the higher or lower religions they may hold simply represent, not always the actual facts of God and of truth, but the degree of spiritual light which the different races have themselves craved for; it is, then, that their little narrow systems so often clash with God's great order. But "Our Father which art in heaven," believed in and uttered by every man with the full consciousness that every other man in the world has an equal right and claim to utter it, is a revelation which never did and never will need to be reconciled to a single fact of science in all this great and wide universe.

**"Medicoes Under the Microscope."**

In common with most of his medical brethren, the writer, a week or two ago, received a printed post-card which announced that certain medical men were to be placed "under the microscope" for the benefit of the readers of the *Gentlewoman*, and that the President of the Royal College of Physicians of London was to be the "lead-off." As an orthodox doctor the recepient felt a little shock on learning that such a departure was in contemplation. As a man of the world, however, he quickly made up his mind that though certain dovecotes might be fluttered, the world would probably still continue to spin round; and he therefore decided that the right course to pursue was to say nothing, and wait the *dénouement*. Punctually, according to promise, the *Gentlewoman* presented its readers with a pen-and-ink sketch of Sir Andrew Clark on January 10th. Of the sketch itself it cannot but be said that it is drawn with a firm and skilful hand, that it is true to life and fact, and that it ought not to offend any susceptibilities, either those of Sir Andrew Clark, of his friends, or of his enemies. One circumstance in the story as there pourtrayed is the fact, somewhat uncommon among doctors, that "with the exception of insignificant contributions to the medical papers, and a brochure or two published in early life, Sir Andrew Clark has added nothing to the medical history of the nineteenth century." We were not aware, until the statement appeared in the *Gentlewoman*, that such was the case; though, of course, we might easily have made the discovery by turning up the "Medical Directory." It will be to many medical readers, as it is to us, a distinct satisfaction to learn that so eminent a physician and so able a man as Sir Andrew Clark has not flooded the world with the products of "scissors and paste." Quite as much as nine-tenths of the so-called medical literature of the times is mere worthless trash, because it consists of nothing else but miserable pilferings by one medical self-advertiser from the books of other medical self-advertisers. It is not considered legitimate for a medical man to advertise himself except in one way: and that is by writing a book. Now, no man who has a single particle of respect for himself or for literature will consent to make the latter a mere vehicle for advertising his own knowledge and skill. Still less will he append his name to a mass of print which he knows in his heart was never designed as a permanent contribution to general or special literature, but only to make him known to other medical men and the world—specially the world. Sir Andrew Clark has never done that kind of thing, it appears; and he is much to be congratulated thereupon. But if there is a time to be silent, there is also a time to speak. Nothing is so valuable to the world, in any department of thought and activity, as the results of experience. Experience—*pace* ye bacteriologists and inoculators—is the very life and soul of medical practice, and always must be so. Sir Andrew Clark has had an almost unparalleled experience. Not only has it been large, but it has been penetrating, shrewd, honest, intelligent. The time has fully come for the rich and ripe fruits of that unique experience to be given to medicine and the world. We venture to urge upon Sir Andrew Clark that he can render to the medicine of Europe and the world the greatest of all the services he has ever rendered to it by telling the story of his life-work. Of his skill with pen and tongue all his friends are well assured. If his fastidiousness makes him lock up his garnered wealth permanently in his own breast, it will be a grave fault. He owes it to the world to give back, with his own mint stamp upon them, the golden stores of knowledge and experience it has so generously enabled him to treasure up.

**A Children's Ailing Home at Aberdeen.**

"The latent recklessness of the Scottish character," is a phrase which the writer read with much amusement, somewhere, recently; though he cannot tell where. It was a piece of keen wit, and could only have been written by a Scotchman. If there be one town in Scotland less "reckless" than another, that town is Aberdeen. Aberdeen has a Children's Hospital, and also a Convalescent Cottage for Children, at Culter, and the Convalescent Cottage is so well managed that Mr. Charles Macdonald, of Froghall, has presented, or is about to present, a large house with a considerable piece of ground in the country to the Children's Hospital for the purpose of forming a country home for ailing children and a convalescent house for the Children's Hospital, both in one. But in spite of these facts a committee has been formed, and an independent scheme is proposed for providing a third children's institution of the nature of a home for the ailing. Now if Aberdeen had been Glasgow with a population of 600,000, or even Edinburgh with nearly 250,000, the addition of a third children's institution might have been a matter of small moment. But in a town of a little over 100,000 inhabitants, with a large general hospital, a well-equipped Children's Hospital, and a Convalescent Institution and Home for Ailing Children already in existence, it seems both a pity and a mistake to multiply agencies and increase expenditure when the present resources are sufficient, or could easily be made sufficient, to meet all needful demands. To lecture Scotchmen on economy and prudence would be a little impertinent, and would be decidedly "carrying coals to Newcastle." But we venture to hope that our hint may be accepted, and that the proposed new scheme may not be adopted without the full and sober consideration of the wisest of the many thoughtful men of Aberdeen.

